# The efficacy and safety profile of biologic therapy with etanercept in juvenile idiopathic arthritis

**DOI:** 10.1186/1546-0096-9-S1-P119

**Published:** 2011-09-14

**Authors:** SA Alfantaki, S Stavrou, A Siamopoulou-Mavridou

**Affiliations:** 1 Rheumatology Unit, Pediatric Clinic, Medical School, University of Ioannina, GR-45500 Ioannina, Greece

## Background

Juvenile idiopathic arthritis (JIA) is the most common rheumatic disease in childhood. The treatment of JIA has been revolutionized in the last decade by the use of novel biologic agents that have much improved patients’ outcomes.

## Aim

To evaluate the efficacy and safety profile of long-term administration of etanercept (ETN) to JIA patients, in combination or not with DMARDs, such as methotrexate.

## Methods

We enrolled 25 patients affected by JIA fulfilling the ILAR criteria and undergoing ETN treatment from 6/1999 to 12/2010, in combination with methotrexate 12 of them. Medical charts were reviewed for physical and laboratory findings. All patients’ clinical status was monitored for functional consequences of JIA by the Steinbrocker’s revised criteria. It was confirmed that all patients had already received BCG vaccination at onset. The mean duration of disease prior to ETN treatment was 10 years while the mean duration of ETN administration was 51 months (range 26-74). All patients were closely monitored for adverse events. ETN was discontinued in 7 patients whose follow-up for a mean period of 15.7 months (range 26-74) was free of relapse.

## Results

Response to ETN was evident in 24 patients, of whom 18 had a very early amelioration of clinical signs and symptoms in the first month. Physical disability was improving throughout the follow-up. Ten patients met the criteria of clinical remission on medication. After withdrawal of ETN the disease remained inactive until now in 7 patients. With regard to safety concerns, no adverse events are to be reported.

## Concusion

Biologic therapy with ETN in JIA seems to be efficient and safe

